# Steroid Sulfatase Deficiency and Androgen Activation Before and After Puberty

**DOI:** 10.1210/jc.2015-4101

**Published:** 2016-03-22

**Authors:** Jan Idkowiak, Angela E. Taylor, Sandra Subtil, Donna M. O'Neil, Raymon Vijzelaar, Renuka P. Dias, Rakesh Amin, Timothy G. Barrett, Cedric H. L. Shackleton, Jeremy M. W. Kirk, Celia Moss, Wiebke Arlt

**Affiliations:** Institutes of Metabolism and Systems Research (J.I., A.E.T., S.S., D.M.O., C.H.L.S., W.A.) and Cancer and Genomic Sciences (T.G.B.), University of Birmingham, Birmingham B15 2TT, United Kingdom; Centres for Endocrinology, Diabetes and Metabolism (J.I., A.E.T., R.P.D., T.G.B., C.H.L.S., J.M.W.K., W.A.) and Rare Diseases and Personalised Medicine (T.G.B.), Birmingham Health Partners, Birmingham B15 2TH, United Kingdom; Departments of Paediatric Endocrinology and Diabetes (J.I., R.P.D., T.G.B., J.M.W.K.) and Paediatric Dermatology (C.M.), Birmingham Children's Hospital National Health Service Foundation Trust, Birmingham B4 6NH, United Kingdom; MRC-Holland bv (R.V.), 1057-DN Amsterdam, The Netherlands; Department of Paediatric Endocrinology (R.A.), Great Ormond St Hospital for Children, London WC1N 3JH, United Kingdom; and Benioff Children's Hospital (C.H.L.S.), University of California San Francisco, Oakland, California 94609

## Abstract

**Context:**

Steroid sulfatase (STS) cleaves the sulfate moiety off steroid sulfates, including dehydroepiandrosterone (DHEA) sulfate (DHEAS), the inactive sulfate ester of the adrenal androgen precursor DHEA. Deficient DHEA sulfation, the opposite enzymatic reaction to that catalyzed by STS, results in androgen excess by increased conversion of DHEA to active androgens. STS deficiency (STSD) due to deletions or inactivating mutations in the X-linked *STS* gene manifests with ichthyosis, but androgen synthesis and metabolism in STSD have not been studied in detail yet.

**Patients and Methods:**

We carried out a cross-sectional study in 30 males with STSD (age 6–27 y; 13 prepubertal, 5 peripubertal, and 12 postpubertal) and 38 age-, sex-, and Tanner stage-matched healthy controls. Serum and 24-hour urine steroid metabolome analysis was performed by mass spectrometry and genetic analysis of the *STS* gene by multiplex ligation-dependent probe amplification and Sanger sequencing.

**Results:**

Genetic analysis showed *STS* mutations in all patients, comprising 27 complete gene deletions, 1 intragenic deletion and 2 missense mutations. STSD patients had apparently normal pubertal development. Serum and 24-hour urinary DHEAS were increased in STSD, whereas serum DHEA and testosterone were decreased. However, total 24-hour urinary androgen excretion was similar to controls, with evidence of increased 5α-reductase activity in STSD. Prepubertal healthy controls showed a marked increase in the serum DHEA to DHEAS ratio that was absent in postpubertal controls and in STSD patients of any pubertal stage.

**Conclusions:**

In STSD patients, an increased 5α-reductase activity appears to compensate for a reduced rate of androgen generation by enhancing peripheral androgen activation in affected patients. In healthy controls, we discovered a prepubertal surge in the serum DHEA to DHEAS ratio that was absent in STSD, indicative of physiologically up-regulated STS activity before puberty. This may represent a fine tuning mechanism for tissue-specific androgen activation preparing for the major changes in androgen production during puberty.

## Abstract (precis)

“Steroid metabolome analysis reveals mild androgen deficiency and compensatorily increased 5alpha-reductase activity in steroid sulfatase (STS) deficiency and upregulation of STS activity in healthy boys before puberty.”

Sulfation has been identified as a critical step in regulating the balance between conversion of the principal androgen precursor dehydroepiandrosterone (DHEA) to active androgens and its inactivation through sulfation to DHEA sulfate (DHEAS) (Figure 1). The latter reaction is mainly catalyzed by DHEA sulfotransferase, SULT2A1, and recent reports have revealed this enzyme as a crucial switch controlling androgen activation. Disruption of DHEA sulfation due to inactivating mutations in the human gene encoding PAPSS2, a crucial cofactor of SULT2A1, has been shown to result in increased androgen activation and a polycystic ovary syndrome (PCOS) phenotype in both homozygous and heterozygous individuals (1, 2).

**Figure 1.**
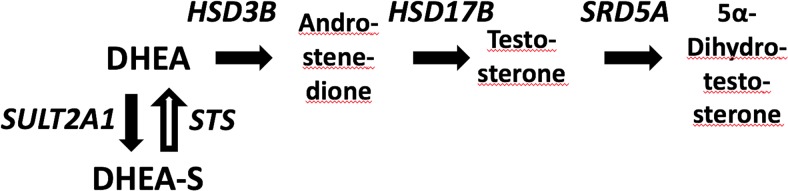
Androgen activation pathway from dehydroepiandrosterone (DHEA), which is either inactivated to DHEA sulphate (DHEAS) or activated via androstenedione and testosterone to the most powerful androgen, 5a-dihydro-testosterone. HSD3B, 3beta-hydroxysteroid dehydrogenase; HSD17B, 17beta-hydroxysteroid dehydrogenase; SRD5A, 5alpha-reductase; SULT2A1, DHEA sulfotransferase; STS, steroid sulfatase.

Consequently, it appears reasonable to assume that a defect in DHEA desulfation catalyzed by the enzyme steroid sulfatase (STS), may result in reduced sex steroid levels. STS also known as aryl sulfatase C, is a membrane-bound microsomal enzyme and member of a highly conserved family of aryl sulfatases. Members of that enzyme family catalyze the cleavage of the sulfate moiety from a variety of substrates, including conjugated steroids and other hormones, proteoglycans, posttranslationally modified proteins and aromatic compounds (for comprehensive reviews, see Refs. 3, 4). STS hydrolyzes the sulfate moiety of sulfated 3β-hydroxysteroids and has a high substrate affinity to DHEAS, the most abundant prohormone in the human circulation.

A previous study in healthy adult men has suggested that STS has no significant impact on systemic androgen reactivation from DHEAS. After iv infusion of DHEAS, participants did not show any increase in circulating concentrations of DHEA or active androgens whereas oral administration of nonsulfated DHEA resulted in significant increases of both DHEAS and active androgens (5).

The *STS* gene is localized on the short arm of the X-chromosome (Xp22.3), which is part of the pseudoautosomal region escaping X-inactivation (6). Genetic abnormalities of the *STS* gene cause STS deficiency (STSD) resulting in the skin condition X-linked ichthyosis (XLI), a common inborn error of metabolism with a reported prevalence of 1:1500 to 1:6000 males (7, 8). STSD/XLI is characterized by thickening of the epidermis with large brown scales of the skin, which are thought to be due to accumulation of sulfated sterols, mainly cholesterol sulfate, in the stratum corneum of the epidermis (9–11).

Only very few studies in men with XLI/STSD have explored sex steroid metabolism but without providing detailed clinical information (12–14), and there is no previous study assessing pubertal development and androgen production at the key developmental stages of adrenarche and puberty in these patients. However, severe androgen deficiency has not been reported yet in XLI/STSD, and infertility is not part of its clinical spectrum, with the exception of patients with larger genomic deletions affecting other genes impacting on reproduction (7).

In this study, we have explored androgen generation and metabolism in a large cohort of boys, adolescents and young men with XLI/STSD with genetically defined mutations confined to the STS locus only, to investigate whether loss of STS function impacts on androgen balance during adrenarche and puberty.

## Materials and Methods

### Patients and study protocol

Inclusion criteria were age between 6 and 30 years and an established diagnosis of XLI/STSD, either based on clinical, biochemical or genetic testing. Exclusion criteria for both patients and sex- and age-matched healthy volunteers were chronic severe disease potentially affecting DHEA secretion (eg, rheumatoid arthritis, ulcerative colitis, cancer), steroid treatment during the preceding 12 months, including steroid inhalers, intake of other drugs known to alter steroid metabolism, and impairment of liver or kidney function due to concomitant disease or medication.

The Warwickshire Research Ethics Committee provided approval of the study protocol. All participants provided written informed consent; in individuals under 18 years, additional assent from at least 1 parent was obtained. On the study day, participants arrived after an overnight fast at the clinical research facility. A clinical history was taken and participants underwent a physical examination, including a dermatological inspection and a detailed assessment of pubertal development including Tanner stages and testicular volume determined by referring to a Prader orchidometer. Anthropometric data (height, weight, body mass index [BMI]) were recorded. Blood samples were obtained for measurement of serum steroid hormones and germline DNA extraction. All participants collected a 24-hour urine for evaluation of the steroid metabolome.

### Genetic analysis

*STS* gene deletions are the underlying abnormality in 90% of patients with XLI. Therefore, we used multiplex ligation-dependent probe amplification (MLPA) as the primary approach to genetic analysis. The MLPA assay was designed by MRC-Holland bv, comprising 11 probes specifically targeting the coding exons of the *STS* gene to identify partial or complete *STS* gene deletions. In addition, the assay also targets the coding exons of the neighboring *KAL1* locus as well as the *HDHD1* locus located centromeric and telomeric from *STS*, respectively. MLPA was performed using standard reaction conditions according to the manufacturer's protocol. In patients with no abnormalities on MLPA analysis, PCR amplification of the entire coding region of the *STS* gene (9 fragments), including intron/exon boundaries from genomic DNA, was performed (for primer sequences see Supplemetal Table 1). Direct sequencing was carried out using an ABI3730 sequencer (Applied Biosystems Inc), and sequencing analysis was carried out using the CLC Main Workbench software (CLC bio).

### Serum and urine steroid measurements

Serum steroids were measured by liquid chromatography-tandem mass spectrometry (LC-MS/MS) employing a Waters Xevo mass spectrometer with Acquity uPLC system as described previously (2, 15). In brief, unconjugated serum steroids (DHEA and testosterone) were extracted from 200 μL of serum via liquid-liquid extraction using methyl tert-butyl ether. After extraction, oxime derivatization of carbonyl groups was performed, a modification that provides enhanced sensitivity of DHEA measurement (16). Tandem MS of the steroid oximes employed electrospray ionization in positive mode.

DHEAS and cholesterol sulfate were extracted from serum in a method adapted form Chadwick et al (17). In brief, 20 μL of serum were taken, and internal standard was added, followed by 20 μL of 0.1mM zinc sulfate for protein precipitation. Subsequently, 100 μL of acetonitrile was added, the sample centrifuged, and 100 μL of the supernatant were transferred to a new plate, evaporated, and reconstituted in methanol/water before LC-MS/MS analysis. Measurement of serum DHEAS and cholesterol sulfate was performed in employed electrospray ionization negative mode.

Steroids were identified and quantified via comparison of retention time and 2 mass transitions (multiple reaction monitoring) to an authentic standard. Each steroid was quantified relative to a deuterated internal standard (testosterone-d3, DHEA-d6, or DHEAS-d2).

Urinary steroid metabolite excretion analysis was carried out by quantitative gas chromatography-mass spectrometry (GC-MS) in selected-ion-monitoring analysis mode, as described previously (18). In brief, urinary steroids were enzymatically released from conjugation, with subsequent recovery of the hydrolyzed steroids by C18 solid phase extraction using Sep-Pak columns (Waters), followed by chemical derivatization before GC-MS-selected-ion-monitoring analysis. We quantified systemic 5α-reductase activity by determining the ratio of the 5α-reduced glucocorticoid metabolite 5α-tetrahydrocortisol (5αTHF) over its 5β-reduced metabolite THF. We also measured urinary DHEA, which represents the sum of urinary DHEA and DHEAS excretion and cannot be distinguished with this method as the sulfate group is removed by a hydrolysis step before the measurement. However, we have found that more than 99% of urinary DHEA originates from DHEAS, ie, proportionate to their respective circulating serum concentrations in the nanomolar and micromolar range, respectively (2). Thus, for clarity, we have labeled the sum of urinary DHEA and DHEAS in results and figures as urinary DHEAS.

### Statistical analysis

A nonparametric test (Mann-Whitney-Wilcoxon) was used to compare the 2 independent parameters from each cohort (control vs STSD). *P* = .05 was assumed to be statistically significant. Data were expressed as median (±interquartile range [boxes] and ±10th–90th percentile [whiskers] ranges). The software GraphPad Prism was used for analysis.

## Results

### Patient characteristics

We recruited 30 patients with STSD and 38 healthy volunteers (controls). STSD patients and controls did not differ with regard to weight standard deviation score (SDS) (controls vs STSD 0.58 ± 1.0 vs 0.31 ± 1.3, *P* = .42), height SDS (0.04 ± 1.2 vs 0.37 ± 1.1, *P* = .92), and BMI SDS (0.66 ± 1.0 vs 0.09 ± 1.4, *P* = .37).

All patients had clinically typical dermatological findings of XLI with different degrees of severity. In most the STSD patients, the diagnosis had been established based on clinical findings only (n = 20). In 7 patients, an additional lymphocyte assay for STS activity had been performed, which had shown decreased activity. Only 3 of 30 patients had previously undergone genetic testing for confirmation of diagnosis.

There was no history of consanguinity in the affected families. We recruited 4 pairs of siblings with STSD and 3 affected brothers from a single family.

Eleven patients had additional conditions, mostly atopic disease such as mild asthma (n = 6), hay fever (n = 4), eczema (n = 1), or multiple allergies (n = 1); 1 patient had been diagnosed with attention deficit hyperactivity disorder.

### Pubertal development

Thirteen STSD patients were clinically prepubertal with testicular volumes less than or equal to 4 mL and prepubertal Tanner stages. Five patients were peripubertal with testicular volumes between 4 and 14 mL; the remaining 12 STSD patients were postpubertal with testicular volumes more than 15 mL and physically fully developed secondary characteristics as assessed by Tanner stages (Table 1).

**Table 1.** Clinical Characteristics of the STSD Cohort

	Patient Number	Age (y)	BMI (kg/m^2^)	Tanner Pubertal Stages			Testicular Volume	
				P	G	A	Left (mL)	Right (mL)
Prepubertal	P01	6.2	13.7	1	1	1	2	2
	P02	6.7	23.0	1	1	1	n.d.	2
	P03	6.9	16.2	1	1	1	2	2
	P04	7.5	15.0	1	1	1	3	3
	P05	7.6	18.1	1	1	1	3	3
	P06	9.1	15.2	1	1	1	2	2
	P07	9.2	14.6	1	2	1	3	3
	P08	9.2	24.1	1	1	1	3	3
	P09	10.0	16.5	1	1	1	3	3
	P10	10.8	16.1	1	1	1	3	3
	P11	11.0	14.6	1	1	1	3	3
	P12	11.1	17.3	1	1	1	2	2
	P13	11.2	23.2	1	1	1	3	3
Peripubertal	P14	11.7	17.5	1	2	1	4	3
	P15	12.8	18.6	2	2	1	4	4
	P16	13.3	16.6	1	2	1	3	4
	P17	13.4	14.8	1	2	1	5	4
	P18	13.4	16.9	1	2	1	5	5
Postpubertal	P19	14.3	19.3	5	5	2	10	15
	P20	15.8	17.8	5	5	2	20	20
	P21	15.8	23.5	5	4	2	20	20
	P22	15.9	20.7	5	5	2	25	25
	P23	16.3	24.3	5	5	2	15	15
	P24	18.0	15.6	5	5	2	20	20
	P25	19.6	25.0	6	5	2	25	20
	P26	20.8	19.3	5	5	2	20	20
	P27	22.9	23.9	6	5	2	20	20
	P28	24.8	25.7	6	5	2	25	25
	P29	26.5	24.9	5	5	2	20	20
	P30	26.6	27.47	6	5	2	20	20

Tanner stages: P, pubic hair; G, genitalia; A, axillary hair; n.d., not detected.

One STSD patient (age 6.7 y) was found to have a unilateral undescended testicle and was subsequently referred for surgical treatment by orchidopexy. No patient had delayed puberty as defined by pubertal onset after the age of 14 years; pubertal progression according to Tanner stages and testicular volumes were appropriate for age in all participants (Table 1).

**Figure 2.**
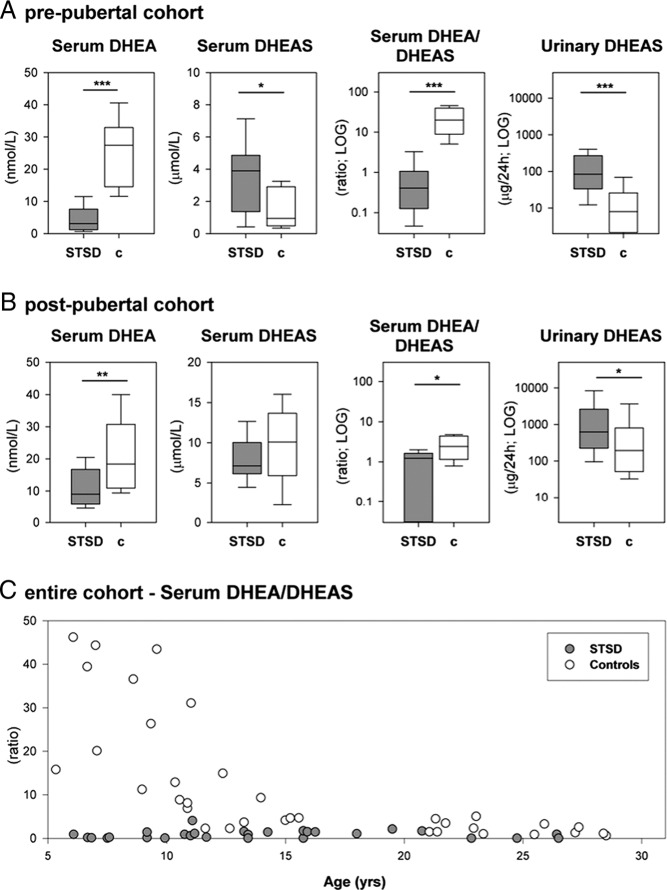
Serum and urinary DHEA and DHEAS in patients with STSD and healthy sex- and age-matched controls. A and B, Levels of serum DHEA and DHEAS, their ratio, reflective of STS activity, as well as the 24-hour urinary excretion of DHEAS. A, Data from the prepubertal subgroup (STSD, n = 13; controls, n = 15). B, Data from the postpubertal subgroup (STSD, n = 12; controls, n = 19). C, Ratio of serum DHEA/DHEAS, reflective of STS activity, is visualized as a function over age (top) (STSD patients; n = 30; gray circles; and healthy controls; n = 38; open circles).

**Figure 3.**
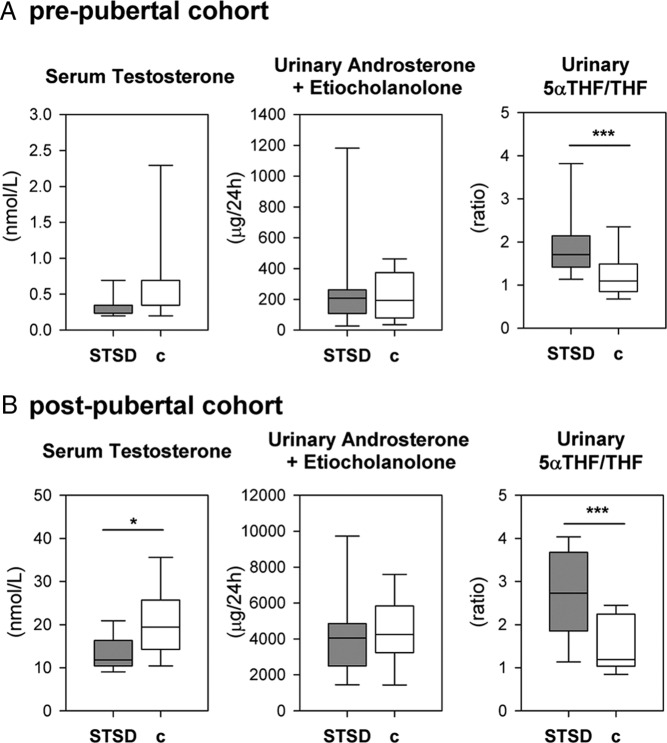
Active androgens and their metabolism of patients with STSD (n = 30) compared with healthy male controls (n = 38). Box and whisker plots depicting median, interquartile range, and 10th–90th percentile are used to visualize serum testosterone (left), the sum of 24-hour urinary excretion of the active androgen metabolites androsterone and etiocholanolone (middle), and the ratio of urinary 5αTHF to THF reflective of net 5α-reductase activity (right). A, Data for the prepubertal subjects (STSD, n = 13; controls, n = 15). B, Results in the postpubertal subgroup (STSD, n = 12; controls, n = 19).

For further analysis, subjects were allocated to 3 subgroups based on their pubertal progression as stated above: prepubertal STSD n = 13, controls n = 15; peripubertal STSD n = 5, controls n = 5; and postpubertal STSD n = 12, controls n = 19. Statistical analysis has been performed in all 3 subgroups (Table 2), but for the purpose of clarity, only the pre- and postpubertal subgroups have been visualized in Figures 2 and 3.

**Table 2.** Median (Interquartile Range) of Serum and Urinary Steroids in STSD Patients and Controls Within the 3 Different Subgroups in STSD Patients (Prepubertal, n = 13; Peripubertal, n = 5; Postpubertal, n = 12) and Healthy Sex- and Age-Matched Controls (Prepubertal, n = 15; Peripubertal, n = 5; Postpubertal, n = 19)

	STSD	Controls	*P* Value
**Serum DHEA (nmol/L)**			
All	6.0 (3.1, 9.1)	20.0 (13.1, 31.7)	<.0001
Prepubertal	6.6 (2.4, 7.6)	20.1 (14.2, 32.8)	<.0001
Peripubertal	5.7 (4.8, 7.4)	11.3 (8.4, 22.3)	.110
Postpubertal	9.0 (6.8, 14.2)	18.4 (11.1, 29.9)	.004
**Serum DHEAS (μmol/L)**			
All	5.1 (4.1, 7.5)	3.5 (1.4, 9.6)	.373
Prepubertal	4.5 (2.0, 5.0)	1.4 (0.7, 3.0)	.014
Peripubertal	6.2 (4.6, 7.1)	3.5 (3.2, 3.7)	.343
Postpubertal	7.1 (6.2, 9.6)	10.1 (6.6, 13.0)	.227
**Serum DHEA to DHEAS ratio**			
All	0.9 (0.1, 1.4)	4.9 (2.3, 16.9)	<.0001
Prepubertal	0.4 (0.1, 1.0)	15.8 (8.5, 36.9)	<.0001
Peripubertal	0.6 (0.2, 1.03)	3.0 (2.3, 6.5)	.029
Postpubertal	1.2 (0.1, 1.6)	2.6 (1.3, 4.5)	.010
**Serum testosterone (nmol/L)**			
All	4.0 (0.4; 11.0)	10.1 (0.4, 17.6)	.145
Prepubertal	0.4 (0.4, 0.7)	0.4 (0.4, 1.22)	.296
Peripubertal	4.0 (1.5, 6.5)	6.3 (2.2, 11.1)	.685
Postpubertal	11.8 (10.4, 15.6)	19.4 (15.3, 25.0)	.008
**Urinary androsterone+etiocholanolone excretion (μg/24 h)**			
All	604 (231, 3526)	780 (235, 4067)	.588
Prepubertal	237 (177, 415)	247 (96, 413)	.957
Peripubertal	466 (226, 567)	456 (256, 1652)	.111
Postpubertal	4055 (2722, 4779)	4193 (3184, 5461)	.521
**Urinary 5αTHF to THF ratio**			
All	2.3 (1.8; 3.2)	1.2 (1.0; 1.7)	<.0001
Prepubertal	1.9 (1.6; 3.1)	1.1 (0.9; 1.3)	.040
Peripubertal	3.2 (3.2; 3.8)	1.2 (1.0; 1.6)	.016
Postpubertal	2.7 (1.9; 3.4)	1.2 (1.1; 2.2)	.003

### Genetic analysis

Genetic analysis confirmed abnormalities within the *STS* gene in all patients. A total of 27 of 30 patients had a complete deletion of the *STS* gene and the adjacent *HDHD1A* gene. There was no evidence for deletion of further coding regions at the Xp22.31/32 locus; in particular, we did not identify *KAL1* gene deletions in any of the included patients (Supplemental Figure 1A). One patient (P16) had a deletion of exon 7 only (Supplemental Figure 1B). In 2 brothers there were no abnormal findings in the MLPA analysis and subsequent Sanger sequencing of the *STS* gene revealed a hemizygous nonsynonymous missense mutation in exon 9 (Supplemental Figure 1C) encoding for p.R454C, a cytosine to thymidine change at position g.114,414 resulting in the replacement of arginine to cysteine on the protein level. This mutation has previously been reported in a patient with XLI (19).

### Steroid metabolome analysis

Serum DHEA was significantly lower in the pre- and postpubertal STSD subjects than in sex- and age-matched healthy controls (Figure 2, A and B). Conversely, serum DHEAS was higher in STSD than in controls, albeit only significantly different in the prepubertal subgroup. However, 24-hour urinary DHEAS excretion in both STSD subgroups was markedly elevated and significantly higher than in controls (Figure 2, A and B).

Of note, the ratio of serum DHEA to DHEAS was significantly higher before puberty in healthy controls (Figure 2C). By contrast the serum DHEA to DHEAS ratio was persistently low in STSD patients of all subgroups (Figure 2C).

Serum testosterone was at age-appropriate low levels in all prepubertal subjects (Figure 3). After puberty, serum testosterone concentrations were significantly lower in STSD patients than in sex- and age-matched controls. However, 24-hour urinary excretion of the 2 major metabolites of active androgens, androsterone and etiocholanolone, did not differ between STSD and controls (Figure 3). Further analysis of the urinary steroid metabolome revealed a significant increase in the ratio of the 5α-reduced glucocorticoid metabolite 5αTHF over THF, indicative of an increase in net 5α-reductase activity and hence an enhanced peripheral androgen activation rate in all subgroups of STSD patients as compared with controls.

## Discussion

Here, we have investigated androgen metabolism and generation and pubertal development in a large, genetically fully characterized cohort of patients with STSD/XLI. We found no gross abnormalities regarding pubertal progression and development in children and adolescents with STSD. Their steroid metabolome was indicative of mild androgen deficiency, with significantly lower circulating concentrations of DHEA and testosterone. However, these appear to be compensated for by enhanced tissue-specific androgen activation including increased net 5α-reductase activity observed in our cohort, resulting in total androgen metabolite excretion rates that did not differ significantly between STSD and healthy controls.

We have genetically characterized the entire cohort and found abnormalities of the *STS* gene in all patients. Complete deletions were detected in 27 of 30 patients, in keeping with previous reports describing deletions as the commonest mutation type in XLI/STSD with an incidence of 80%–90% (6, 20). Importantly, deletions of the neighboring *KAL1* gene resulting in Kallmann syndrome with anosmic hypogonadotropic hypogonadism were not present in this cohort. Similarly, other forms of contiguous gene deletion syndromes frequently described in patients with XLI (21, 22), were not detected, hence the changes we found can only be associated with the loss of *STS* gene function. All our patients with complete *STS* deletions had a concurrent deletion of the *HDHD1* gene upstream of the *STS* locus, which is a previously reported feature in most patients with complete *STS* deletions (23, 24). *HDHD1* encodes for a pseudouridine-5′-phosphatase involved in RNA metabolism, and its molecular function has been described in detail only recently (24). Haloacid Dehalogenase-like Hydrolase domain-containing 1 (HDHD1) dephosphorylates pseudouridine 5′-phosphate, a modified RNA nucleotide present in tRNAs, rRNAs, and small nuclear RNAs; pseudouridine is excreted in urine and serves as a biomarker for certain cancers (25). The importance of HDHD1 in normal physiology is not well understood; an *Hdhd1* knockout mouse has not yet been reported. One can speculate that HDHD1 contributes to some phenotypic features observed in patients with XLI; however, detailed genotype-phenotype studies are lacking partially due to insufficient genetic characterization of XLI patients of earlier cohorts. Preumont et al (24) have previously speculated that the absence of pseudouridine 5′-phosphatase activity may contribute to the development of testicular cancer and cryptorchidism as this has not been observed in patients with missense mutations or partial deletions of the *STS* gene. The one patient with an undescended testis in our cohort also had a *HDHD1* deletion. However, at present there is no conclusive evidence for functional involvement of HDHD1 in testicular descent.

One patient in our cohort had a partial deletion of exon 7 of the *STS* gene. Although partial *STS* deletions have been reported in XLI (26, 27), the deletion of exon 7 appears to be a novel finding. In addition, 2 brothers in our study carried the known p.R454C missense mutation, previously described in a patient with XLI; in vitro functional analysis employing radiolabeled ^3^H-DHEA assays with patient leukocytes demonstrated reduced STS activity (19).

Pubertal development and progression was not abnormal in this cohort of STSD patients; Tanner stages, testicular volumes and height SDS did not differ from controls. The onset of pubertal changes reported by the STSD patients in our cohort was between 11 and 11.5 years, which is considered to be within the normal range of pubertal onset in males (28, 29). However, our cohort included 5 boys with STSD who were clinically at the beginning of puberty according to their testicular volume and Tanner stages. Of those, 3 had not developed any pubic hair by the age of 13, which is somewhat late. To investigate in more detail whether the onset of pubertal development in STSD boys is normal or delayed would require the prospective observation of a cohort of prepubertal individuals with STSD. None of our STSD patients had children at the time of study participation. However, we have obtained pedigrees in 22/30 STSD patients, which showed a classical x-linked recessive inheritance of XLI, with maternal grandfathers and great-grandfathers affected by XLI. This, in line with previous studies in patients with XLI (for review, see Ref. 7), suggests that infertility is not part of the spectrum in STSD.

**Table 3.** Summary of Previous Studies Investigating Circulating Steroid Concentrations in Patients With STSD

Study	Subjects	Findings in Blood Samples	Steroid Quantification by
Sánchez-Guijo et al (10)	12 STSD, 19 controls adult age	Increased levels of cholesterol sulfate, 16-OH-DHEAS, DHEAS, androstenediol sulfate, androsterone sulfate, DHT sulfate, and progestin sulfates in STSD	LC-MS/MS
Delfino et al (14)	33 STSD, 33 controls age 3–70 y	DHEAS increases during puberty but not significantly; cholesterol sulfate persistently elevated	GC-MS
Milone et al (13)	15 STSD, 15 controls age 22–33 y	DHEAS elevated when measured with GC-MS, no difference found with RIA	GC-MS and RIA
Ruokonen et al (40)	6 STSD, 6 controls adult age	Normal testosterone and LH; significantly increased sulfated pregnenolone, 17-hydroxypregnenolone, dehydroepiandrosterone, and 5-androstene-3β,17β-diol and decreased corresponding unconjugated steroids; increases in testosterone, 17-hydroxyprogesterone, and estradiol were similar in STSD and controls after hCG stimulation	RIA
Lykkesfeldt et al (12)	20 STSD (age 20–60 y) 100 controls (age 20–70 y)	Nonsignificant trend towards higher DHEAS in STSD; no decline with age; lower levels of androstenedione and 17β-estradiol in STSD; higher LH in STSD	RIA
Muskiet et al (41)	7 STSD, 20 controls adult age	DHEAS normal in 6 of 7 STSD patients	GC-MS
Epstein et al (11)	7 STSD patients	Cholesterol sulfate persistently increased in all subjects, DHEAS frequently but not consistently high	GC-MS
Ruokonen et al (42)	5 STSD, 10 controls adult age	DHEAS, pregnenolone sulfate, and 5-androstene-3β, 17β-diol sulfate not significantly higher in STSD	RIA

Previous studies on steroid production in STSD have mostly focused on cholesterol sulfate and DHEAS; the latter was not consistently reported as elevated, perhaps due to the different methodologies employed for steroid quantification (Table 2). Our study is the first to employ 24-hour urine steroid metabolome analysis by GC-MS and serum steroid analysis by LC-MS/MS to comprehensively analyze androgen generation and metabolism in STSD. Previously, the most detailed information was available from an adult Danish cohort of 20 XLI patients (12). Although no detailed information on physical and pubertal development was provided, the investigators found biochemical evidence of mild androgen deficiency with increased levels of DHEAS, suggesting an effect of STS on peripheral sex steroid activation. In addition, LH was higher in STSD than in 40 matched controls, suggesting compensatory activation of the hypothalamic-pituitary-gonadal axis. Our study is the first to cover the complete age range from prepuberty to young adulthood with detailed information on pubertal phenotype and genotype. The latter enabled us to exclude any effect of contiguous gene deletions on the observed changes in steroid concentrations. Our results in children and adolescents with STSD are in keeping with those in the Danish adult cohort (12), indicating the presence of mild androgen deficiency.

Urinary steroid metabolome analysis in our STSD cohort demonstrated normal overall androgen metabolite excretion despite lower concentrations of DHEA available for peripheral androgen inactivation. Our finding of enhanced 5α-reductase activity, which leads to increased activation of testosterone to 5α-dihydrostestosterone, suggests the presence of a compensatory mechanism counteracting a slightly lower androgen production rate. Increased 5α-reductase activity is frequently observed in androgen excess conditions like adult PCOS (30–33), but not in children with premature adrenarche (34). Stewart et al proposed the hypothesis that increased 5α-reduction in PCOS comes first in a pathophysiological chain of events leading to enhanced hepatic cortisol clearance, with compensatory hypothalamic-pituitary-adrenal axis activation and enhanced adrenal androgen secretion (30). The underlying cause for the increase of 5α-reductase activity, either a priori or as a secondary event, is still unknown.

Our study is the first to provide information on serum DHEA concentrations in STSD, which has not been measured in any of the previous studies (Table 2) and the results in our cohort demonstrate low DHEA and increased DHEAS. Previous studies indicate minimal contribution of STS to reactivation of DHEAS to DHEA in adults (5) and a dissociation of DHEA and DHEAS is rarely observed (35). Results in our STSD cohort reveal a small but detectable impact of disrupted STS activity to circulating DHEA and DHEAS levels. However, the results from this study do not suggest that patients with STSD require routine endocrine follow-up as they seem to progress through puberty without significant abnormalities.

We also report, for the first time, a significantly increased ratio of serum DHEA over serum DHEAS in healthy prepubertal children that decreases steeply after puberty. This prepubertal increase in the DHEA to DHEAS ratio is absent in STSD patients, indicating that this previously unknown phenomenon is due to a prepubertal, physiological increase in STS activity. Little is known about normal physiological regulation of STS, with some evidence of cytokine-mediated changes in STS expression and activity (36, 37). In addition, it is possible that cofactors of STS could exhibit age- and sex steroid-dependent changes. Sulfatase modifying factor 1 is responsible for the posttranslational modification of a highly conserved cysteine residue to a unique formylglycine residue in the active site of the STS enzyme that is crucially required for catalytic activity; changes in sulfatase modifying factor 1 impact on STS activity with human inactivating mutations resulting in multiple sulfatase deficiency (38, 39).

In summary, we have demonstrated by steroid metabolome analysis that the impact of STSD on androgen generation is small, resulting in only mild androgen deficiency that is apparently compensated for by up-regulation of peripheral androgen activation. This suggests that the sulfotransferase reaction inactivating DHEA to DHEAS is the predominant switch regulating the balance between DHEA activation and inactivation, with significant clinical implications (1, 2). We found evidence for a dissociation of DHEA and DHEAS before puberty in healthy controls that was absent in STSD, suggestive of significantly increased STS activity during the prepubertal period in healthy individuals. We suggest that this represents a mechanism to fine tune tissue-specific androgen action, priming the body for the subsequent changes in gonadal androgen production during puberty.
